# Supplementary material for: Identification and functional analysis of non-coding regulatory small RNA FenSr3 in Bacillus amyloliquefaciens LPB-18
Source: PeerJ. 2023 May 15;11:e15236. doi: 10.7717/peerj.15236 (PMC10194069; doi:10.7717/peerj.15236)
Supplement: Supplemental Information 4 [file peerj-11-15236-s004.zip › KO/CK-vs-T1_map/map04371.html]

KEGG PATHWAY: Apelin signaling pathway - Reference pathway


|  |  |
| --- | --- |
| **Apelin signaling pathway - Reference pathway** |  |

[
Pathway menu
| Organism menu
| Pathway entry
| Show description
| User data mapping
]

Apelin is an endogenous peptide capable of binding the apelin receptor (APJ), which was originally described as an orphan G-protein-coupled receptor. Apelin and APJ are widely expressed in various tissues and organ systems. They are implicated in different key physiological processes such as angiogenesis, cardiovascular functions, cell proliferation and energy metabolism regulation. On the other hand, this ligand receptor couple is also involved in several pathologies including diabetes, obesity, cardiovascular disease and cancer.

|  |  |  |
| --- | --- | --- |
| Reference pathway | 184% 150% 122% 100% 82% 67% 55% | 图片下载 |
